# Survival and functional outcome following endovascular thrombectomy for anterior circulation acute ischemic stroke caused by large vessel occlusion in Sweden 2017–2019–a nationwide, prospective, observational study

**DOI:** 10.1177/15910199211073019

**Published:** 2022-01-19

**Authors:** Teresa Ullberg, Mia von Euler, Johan Wassélius, Per Wester, Fabian Arnberg

**Affiliations:** 1 Neurology, 156327 Department of Clinical Sciences Lund, Lund University, Lund, Sweden; 2 Neurology, 59564 Skåne University Hospital Lund/Malmö, Lund, Sweden; 3 School of Medicine, Örebro University, Örebro, Sweden; 4 Diagnostic Radiology, Department of Clinical Sciences Lund, 156327 Lund University, Lund, Sweden; 5 Neuroradiology, Skåne University Hospital, Lund, Sweden; 6 Department of Public Health and Clinical Science, Umeå University, Umeå, Sweden; 7 Department of Clinical Science, Karolinska Institute Danderyds hospital, Stockholm, Sweden; 8 Department of Clinical Neuroscience, Karolinska Institute, Stockholm, Sweden; 9 Department of Neuroradiology, 59562 Karolinska University Hospital, Solna, Sweden

**Keywords:** Reperfusion, ischemic stroke, acute stroke therapy, registry, survival, functional outcome

## Abstract

**Background:**

Endovascular thrombectomy (EVT) is standard of care for anterior circulation acute ischemic stroke (AIS) caused by large vessel occlusion (LVO), but data on nationwide performance in routine healthcare are sparse. The study aims were to describe EVT patients with LVO AIS, analyze mortality and functional outcome, and compare results with randomized controlled trials (RCTs).

**Methods:**

Data from the Riksstroke and the Swedish Endovascular Treatment of Acute Stroke Registry (RSEVAS) on pre-stroke independent patients, with LVO AIS in 2017–2019, defined as occlusion of the intracranial internal carotid artery, or the M1 or M2 segments of the middle cerebral artery, and groin puncture <6 h of onset, were compared to aggregated HERMES collaboration RCT data. We assessed 90-day survival and function, defined by the modified Rankin Scale. Specific analyzes were stratified by occlusion location.

**Results:**

In all, 1011/2560 of RSEVAS patients matched RCT inclusion criteria. Compared with RCT data, patients were older (73 vs. 68), fewer received intravenous thrombolysis (63.1% vs. 83%), and M2 occlusions were more common (24.5% vs. 8%). 90-day survival in RSEVAS was 85.3%, 42.8% achieved good outcome and 5% had symptomatic intracerebral hemorrhage (sICH). Corresponding outcomes in RCT data were 84.7% survival, 46% good outcome, and 4.4% sICH. Functional outcome was most favorable following M2 occlusions.

**Conclusions:**

EVT patients from our large real-world national dataset differed from RCT patients in several baseline factors including distribution of vascular occlusion site. However, the overall outcome of EVT in our Swedish cohort appeared to well match the pivotal trial findings.

## Introduction

In the wake of several ground-breaking randomized controlled trials (RCTs), endovascular thrombectomy (EVT) has become the new standard of care for acute ischemic stroke (AIS) with anterior circulation large vessel occlusion (LVO).^1–5^ The 2018 Guidelines for Stroke Care issued by the Swedish National Board of Health and Welfare, introduced EVT for anterior circulation LVO AIS within 6 h of stroke onset as a central recommendation.^6^ However, the national implementation of EVT had preceded the recommendation with all six Swedish comprehensive stroke centers already to various extent performing EVT treatment.

EVT performance in the Swedish routine clinical setting has not yet been evaluated, and there are few other reports on how well results from clinical trials can be translated into routine healthcare on a nationwide scale.^7–10^ The Swedish Stroke Register (RS)^11^ and the Swedish EndoVAscular Treatment of Stroke Register (EVAS)^12^ jointly (RSEVAS) provide necessary and sufficient data to perform a nationwide real-world assessment of EVT.

## Aims

The aims of this study were to (1) describe the nationwide Swedish EVT patient cohort with anterior circulation AIS caused by LVO in the intracranial internal carotid artery (ICA), or the M1 or M2 segments of the middle cerebral artery (MCA), (2) evaluate clinical outcomes, (3) and to compare results to aggregated randomized clinical trial data from the HERMES (Highly Effective Reperfusion Evaluated in Multiple Endovascular Stroke Trials) collaboration.^13^

## Methods

### Study population

All patients ≥18 years (1) registered in both RS and EVAS registries during 2017–2019 (2) who were pre-stroke independent defined as modified Rankin scale (mRS) score 0–2 AND (3) had groin puncture within six hours from symptom onset AND (4) anterior circulation LVO defined as either the intracranial ICA, or the M1 or M2 segment of the MCA, were included for analysis and compared to a meta-analysis using pooled data (EVT arm only) from five randomized trials (the HERMES collaboration) published in 2016.^13^ These trials were heterogenous regarding patient selection. We used a pragmatic approach and applied no upper age limit, included patients with ongoing anticoagulant treatment irrespective of international normalized ratio (INR) value, and included M2 occlusions.

### Data sources

#### The Swedish stroke register – RS

RS is the Swedish quality register for stroke care covering 90% of Swedish hospitalized stroke patients, and collecting data on demographics, pre-stroke function, vascular risk factors, as well as variables of stroke care and treatments.^11^ At three months post-stroke, self-reported functional outcome is collected. RS continuously attains data on mortality status from the Swedish Causes of Death Register (>99% coverage).

#### Swedish EndoVAscular Treatment of Stroke Register – EVAS

EVAS is the Swedish quality register for endovascular stroke treatment capturing detailed procedural and technical data as well as early radiological and clinical outcomes.^12^ Since its start in 2014, the number of thrombectomies for AIS has increased, and register coverage in recent years has been approximately 90%.

#### The merged RSEVAS database

In 2020, RS and EVAS databases were merged into RSEVAS, resulting in a nationwide database of EVTs performed in Sweden.

### Main variables

Intracranial occlusion location was identified on digital subtraction angiography (DSA) and defined by its proximal end. Tandem occlusion was defined as occlusion of the extracranial ICA in combination with an LVO, and isolated extracranial ICA occlusions were not included. Process times: onset to needle (OTN), door to needle (DTN), onset to groin puncture (OTG), onset to revascularization (OTR) time, and groin puncture to revascularization (GTR) were calculated from source data. Intravenous thrombolysis (IVT) was with alteplase in a majority of cases (95.9%), and in 4.1% with tenecteplase. The two drugs were grouped. Degree of recanalization was estimated using the modified Treatment In Cerebral Infarction (mTICI) score (0–3), with successful recanalization defined as mTICI 2b­–3, and excellent recanalization as 2c–3. Symptomatic intracerebral hemorrhage (sICH) was defined as presence of any parenchymal hemorrhage on follow-up brain imaging with a deterioration in National Institutes of Health Stroke Scale (NIHSS) score of ≥4 points. Complications in EVAS are defined as either procedure-related or non-procedural (see Supplementary Table 1).

In RS, functional status at 90 days across the mRS is estimated using a validated algorithm based on variables on dressing, toileting, mobility, living situation and need of help or support from next of kin. Functional independence was defined as mRS 0–2.

Missing data included patients not returning the follow-up questionnaire and missing or incomplete data for individual variables in registered patients.

### Statistics

IBM SPSS 25 was used. Categorical variables were summarized as proportions and compared with χ^2^ test. Quantitative variables were presented as medians and compared using Kruskal Wallis tests. Kaplan-Meier life tables were used to calculate probability of death. Log rank test was used to compare mortality between groups. Patients who were alive but lost to 90-day follow-up were omitted from analysis and functional outcome data from followed up survivors were extrapolated to those lost to follow-up. We also displayed functional outcome across the mRS including missing as a separate category. Multiple imputation techniques were not used.

Concurring conditions registered in RS; hypertension, diabetes, atrial fibrillation and previous stroke/TIA were used to create a sum variable to estimate comorbidity burden with categories of 0, 1, 2, and 3–4 and each condition had equal weight (1).

## Results

### Included patients, baseline characteristics and procedural data

In 2017–2019, 2560 patients were registered in RSEVAS and 39% (1011; 2017 n = 280, 2018 n = 338, 2019 n = 393) fulfilled the HERMES inclusion criteria, without significant differences between the years. Reasons for non-inclusion of 60.5% (n = 1549) of EVT patients were: OTG >6 h (34%, n = 870), pre-stroke dependency (13%, n = 332), and other sites of occlusion; vertebrobasilar, anterior cerebral artery, or distal MCA (M3/M4) occlusion (in all 28.6%, n = 732). Some fulfilled >1 criterion for non-inclusion.

Baseline characteristics in RSEVAS and HERMES are shown in Table 1. Median age in RSEVAS (73) was higher than in HERMES pooled data (68). OTN and OTR compare well. M2 occlusions were more common in RSEVAS (24.5% vs. 8%).

**Table 1.** Baseline characteristics, procedural, and safety outcome data in RSEVAS and HERMES EVT arm. Missing data were <2%, except for smoking (18%). HERMES data were reproduced from publication.^13^

Variable	RSEVAS 2017–2019 matching HERMES n = 1011 n(%)	HERMES EVT arm n = 634 n(%)
Demographic characteristics		
Median age (IQR)	73 (65–80)	68 (57–77)
Female sex	463 (45.8%)	304 (48%)
Pre-stroke function		
mRS 0–2	1011 (100%)	634 (100%)
mRS 3–5	0 (0%)	0 (0%)
Vascular risk factors		
Hypertension	592 (58.6%)	352 (56%)
AF total	461 (45.6%)	209 (33%)
previously diagnosed AF	290 (28.7%)	
AF diagnosed at hospital stay	171 (16.9%)	
Diabetes	163 (16.1%)	82 (13%)
Current smoking	103 (10.2%)	194 (31%)
Previous stroke	117 (11.6%)	-
Previous TIA	62 (6.1%)	-
Comorbidity burden (hypertension, diabetes, AF, previous stroke/TIA)		
0	229 (22.7%)	-
1	345 (34.1%)	-
2	286 (28.3%)	-
3–4	151 (14.9%)	-
Clinical characteristics		
Median NIHSS score (IQR)	16 (11–20)	17 (14–20)
Imaging characteristics		
Intracranial occlusion location		
ICA (intracranial ICA / T-occlusion)	197 (19.5%)	133 (21%)
M1, MCA	566 (56.0%)	439 (69%)
M2, MCA	248 (24.5%)	51 (8%)
Other	-	11 (2%)
Treatment details and process times		
Ongoing anticoagulant treatment	180 (17.8%)	-
IVT	620 (61.3%)	526 (83%)
OTN (median, IQR)	95 (74–125)	100 (75–133)
DTN (median, IQR)	29 (18–44)	-
OTG (median, IQR)	187 (137–253)	-
OTR (median, IQR)	245 (187–316)	285 (210–362)
Sucessful reperfusion (mTICI 2b–3)	851 (85.2%)	402 (71%)
Death and adverse events		
sICH	50 (5%)	28 (4.4%)
Death at 90 days	149 (14.7%)	97/133 (15.3%)
Functional outcome at 90 days		
mRS 0–2	338 (33.4%)	291/633 (46%)

IQR: interquartile range, mRS: modified Rankin Scale, AF: atrial fibrillation, NIHSS: National Institute of Health Stroke Scale, ICA: internal carotid artery, MCA: middle cerebral artery, IVT: intravenous thrombolysis, sICH: symptomatic intracerebral hemorrhage, OTN: onset to needle, DTN: door to needle, OTG: onset to groin puncture, OTR: onset to revascularization.

Use of IVT was considerably lower in RSEVAS than in HERMES (61.3% vs. 83%). The number of patients administered IVT in RSEVAS was 605 (61.3%). Reasons for non-treatment (n = 379) were medical contraindications including anticoagulant treatment (71.8%), > 4.5 h since onset (6.3%), mild symptoms (2.4%), intracranial hemorrhage (0.8%), severe symptoms (1.6%), other reasons (17.2%). The proportion of patients administered TPA decreased with increasing comorbidity burden from 73.4% in patients with no concurring conditions, to 43.3% in patients with 3–4 conditions (p < 0.001) (Supplementary Figure 1).

### Baseline and procedural data by occlusion location

Baseline, procedural and safety variables by occlusion location are shown in Table 2. Occlusion locations were 197 (19.5%) intracranial ICA, 566 (56%) M1 occlusions, and 248 (24.5%) M2 occlusions. Median NIHSS in RSEVAS differed significantly by occlusion location (p < 0.001). Tandem occlusions occurred in 102 patients (10.1%) and were more often associated with distal ICA occlusions than with M1 or M2 occlusions. Carotid stenting was used in 42.2% (43/102) with tandem occlusion. sICH did not differ by occusion location but death at 7 days was more common in ICA occlusion (10.4%, M1 5.9%, M2 3.6%, p < 0.007). Procedure-related complications occurred in 7% and did not differ by occlusion location. Medical non-procedural complications occurred in 27.7% and were most common in ICA occlusions (Table 2).

**Table 2.** Comparison of characteristics, procedural data and safety by occlusion location. Missing data were <2%, except 24-h NIHSS (13%) and smoking (18%).

Variable	ICA n = 197 n (%)	M1 n = 566 n (%)	M2 n = 248 n (%)	p-value
Demographic characteristics				
Median age (IQR)	74 (67–81)	73 (65–80)	74 (66–80)	0.294
Female sex	83 (42.1%)	280 (49.5%)	100 (40.3%)	0.028
Vascular risk factors				
Hypertension	116 (59.2%)	331 (58.6%)	145 (58.5%)	0.986
AF	84 (42.6%)	253 (44.8%)	124 (50.0%)	0.249
Diabetes	33 (16.8%)	89 (15.7%)	41 (16.6%)	0.914
Clinical characteristics				
Median NIHSS score (IQR)	19 (14–21)	16 (11–20)	12 (7–18)	<0.001
Treatment details and process time				
IVT treatment	124 (62.9%)	331 (58.7%)	165 (66.8%)	0.083
Tandem occlusion	44 (22.3%)	48 (8.5%)	10 (4.0%)	<0.001
General anesthesia	67 (34%)	193 (34.1%)	66 (26.2%)	0.092
Access				0.491
Femoral	192 (98.0%)	550 (97.2%)	244 (98.8%)	
Radial	0 (0%)	3 (0.5%)	0 (0%)	
Common carotid	4 (2.0%)	12 (2.3%)	3 (1.2%)	
OTG (median, IQR)	178 (129–238)	192 (140–260)	184 (137–248)	0.271
OTR(median, IQR)	242 (191–324)	246 (187–316)	245 (185–317)	0.873
Endovascular therapy				
EVT technique				0.021
DAC	39 (19.8%)	121 (21.4%)	41 (16.5%)	
Stent retriever	151 (76.6%)	428 (75.6%)	187 (75.4%)	
Only attempted EVT	5 (2.5%)	13 (2.3%)	18 (7.3%)	
Other	2 (1.0%)	4 (0.7%)	2 (0.8%)	
Early neurological outcome				
24 h-NIHSS (median, IQR)	6 (1–13)	8 (2–15)	5 (1–11)	0.014
Safety outcomes and adverse events				
sICH	11 (5.8%)	29 (5.2%)	10 (4.1%)	0.691
Death at 7 days	20 (10.2%)	28 (4.9%)	9 (3.6%)	0.007
Procedure-related complication	11(7.2%)	24 (5.8%)	18 (9.6%)	0.246
Non-procedural complication	57 (37.3%)	110 (26.6%)	42 (22.3%)	0.007

IVT: intravenous thrombolysis, IQR: interquartile range, AF: atrial fibrillation, NIHSS: National Institute of Health Stroke Scale, DSA: digital subtraction angiography, ICA: internal carotid artery, MCA: middle cerebral artery, sICH: symptomatic intracerebral hemorrhage, mTICI: modified treatment in cerebral infarction, OTG: onset to groin, OTR: onset to revascularization, DAC: direct aspiration catheter.

**Figure 1.**
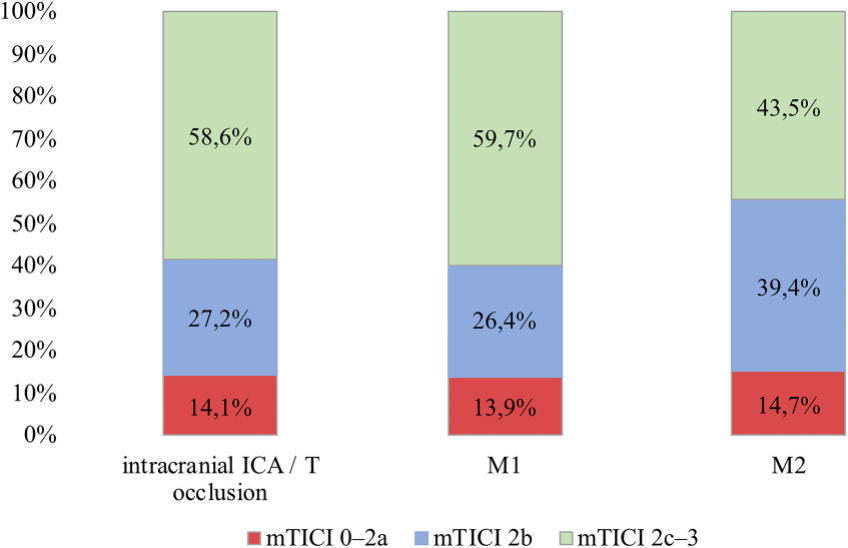
Degree of revascularization according to the mTICI scale by occlusion location.

Successful recanalization (mTICI 2b–3) was achieved in 85.3% (851/998) without differences between occlusion locations. However, excellent recanalization was achieved significantly less often in M2 occlusions (ICA 58.6%, M1 59.7%, M2 43.5%, p < 0.001) (Figure 1). NIHSS at 24 h (captured in 87%) was 6 (1–13), (ICA: 8 (2–15), M1: 6 (2–13), M2: 5 (1–11), p = 0.014).

### Survival at 90 days

Cumulative 90-day survival was 85.3% (n = 842/987) (Supplementary Figure 2(a)). Although a trend towards lower survival in ICA occlusion, there was no significant difference relating to occlusion location (Supplementary Figure 2(b)).

### Loss to follow-up

A total of 189 patients (18.7%) had missing or incomplete data on functional status. Baseline and early outcome data between followed up and not followed up patients are displayed in Supplementary Table 2. The only significant difference was that sICH was more common in not followed up (8% (15/189) versus 4.4% (35/822), p = 0.041).

### Functional outcome at 90 days

**Figure 2.**
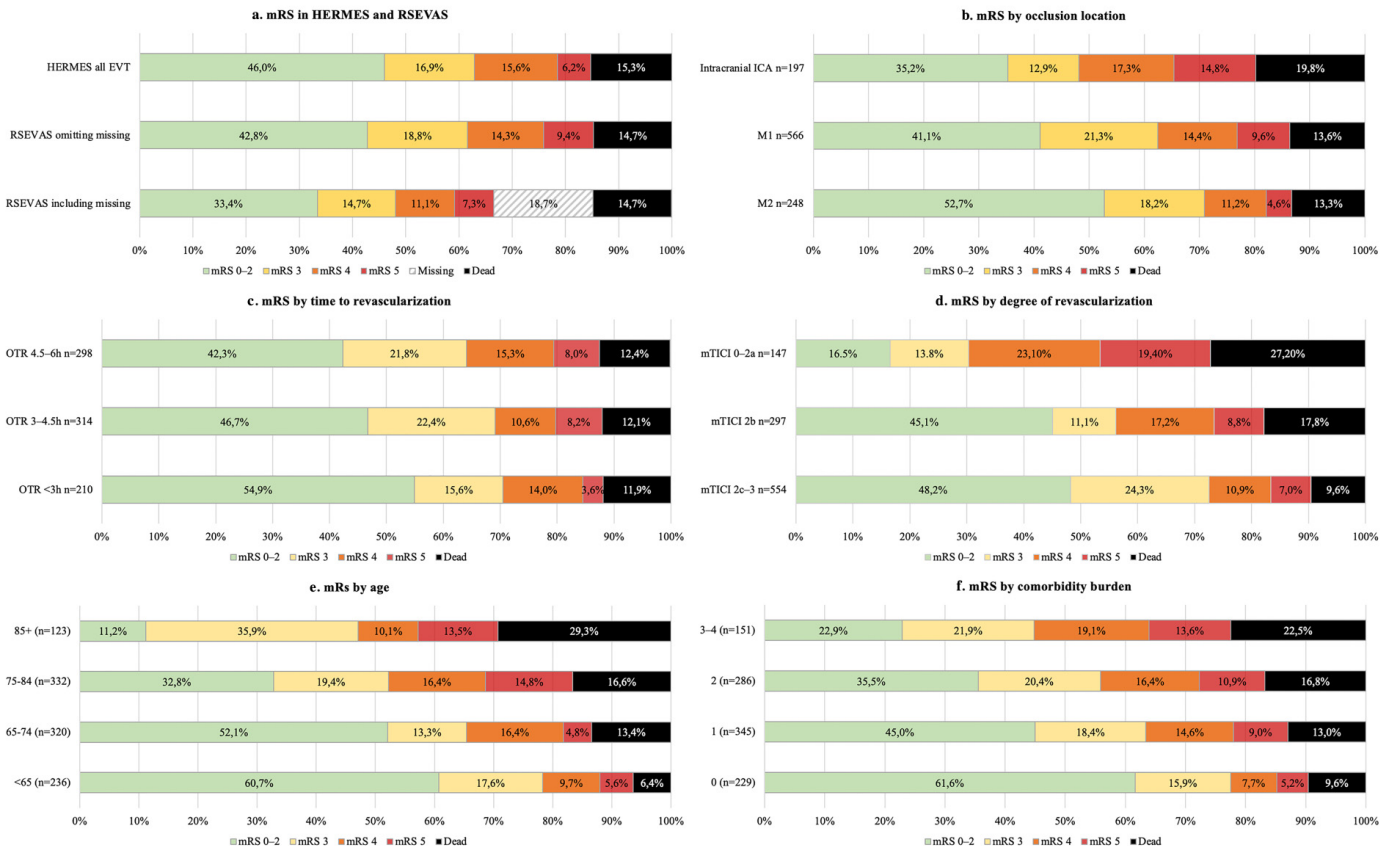
Distribution on the modified Rankin Scale: (a) for all patients in RSEVAS and the HERMES EVT arm (13), (b) by occlusion location, (c) by time to revascularization, (d) by degree of revascularization, (e) by age, (f) by comorbidity burden.

In all, 822 (81.3%) patients were followed up at 90 days. Data on mortality status were complete. Patients lost to follow-up (n = 189) were alive but with unknown functional status. Estimated functional outcome across the mRS, in RSEVAS and HERMES, is shown in Figure 2(a). Good outcome (mRS 0–2) was achieved in 42.8% (33.4% (338/1011) when including missing). Outcome in RSEVAS differed significantly by occlusion location with the most favorable outcomes in M2 occlusions (p = 0.001) (Figure 2(b)). Shorter time from onset to recanalization was associated with significantly better outcomes (Figure 2(c)), but mRS outcome in patients with successful reperfusion (mTICI 2b-3) did not differ significantly by time to revascularization in bivariate analysis (Figure 2(d)). Increasing age and comorbidity burden were also associated with poor outcomes (Figure 2(e)–(f)) (p < 0.001). Data including missing at 90 days are shown in Supplementary Figure 3(a)–(e).

## Discussion

We present real-world nationwide data on anterior circulation AIS caused by LVO in pre-stroke independent patients treated with EVT within six hours of symptom onset. In the studied time period of 2017–2019, Swedish guideline recommendations were limited to this patient group (and to patients with basilar artery occlusion accounting for only 7.8%, 200/2560 of EVTs, data not shown),^6^ yet they accounted for only 39.5% of all EVT patients. Not until 2020 were the Swedish guidelines extended to cover the extended time window of up to 24 h, speaking to the fact that clinical practice often precedes official recommendations. Performance compares well to RCTs (HERMES collaboration),^13^ and to data from similar registers^7,9^ with regards to technical success rate, survival, functional independence on follow-up and safety outcomes. In RSEVAS, the rate of functional independence at 90 days was 42.8%, all-cause mortality at 90 days was 14.7%, and sICH was reported in 5%.

M2 occlusions accounted for only 8% in the 2016 HERMES meta-analysis (4%–14% in the individual five trials), while representing almost a fourth (24.5%) of our cohort. In fact, most M2 occlusions in HERMES were mostly wrongly classified as M1 occlusions and may not be representable for M2 occlusions in general.^13^ The MCA, being the phylogenetically youngest cerebral vessel, developed from the lenticulostriate system with the expansion of the neocortex.^14^ Recognizing the MCA as a branch of lenticulostriate vessels explains its various branching patterns, but also explain the difficulties in classifying MCA occlusions by its segments. In our as well as in other cohorts, the registration of MCA occlusion location may not be entirely congruent. In RSEVAS, M2 occlusions had significantly lower median NIHSS score compared to M1 or ICA occlusions, indicating inclusion of both dominant and non-dominant M2 segment occlusions, which may explain their better prognosis. However, median NIHSS across all patients differed little between RSEVAS and HERMES (16 vs. 17 points). Excellent recanalization was achieved less often in M2 occlusion, a result not seen in other studies.^15,16^ Despite that, outcomes were more favorable. There was a significant difference in EVT technique used with less use of DAC's in M2 occlusions, but otherwise treatment characteristics or procedure-related complication rate did not differ between groups.

There are no randomized trials exclusively investigating M2 EVT, but in a recent meta-analysis from the HERMES collaboration published in 2019 and including 130 patients, results favored EVT over medical treatment in the M2 subgroup.^17^ Good outcome in observational studies^18,19^ and in the HERMES 2019 subgroup analysis^17^ ranged from 45.5%–59.3%, 90-day mortality ranged from 12%–24.3%, while reported sICH ranged from 0%–6.6%. This is in good agreement with our finding of 52.9% good outcome, 13.3% 90-day mortality, and 4.1% sICH in nationwide data. Even though many guidelines restrict their recommendations to proximal LVOs,^20,21^ most neurointerventionalists routinely offer EVT to patients with M2 occlusions.^22^

The high recanalization rate (84%) is translated into functional independence in only less than half of the patients with mTICI 2b–3. This mismatch is seen in similar studies,^7,9^ as well as in HERMES,^13^ and indicates irreversible damage to the brain by the time recanalization is achieved. Re-occlusion may also explain the mismatch in a minority of cases. We demonstrate a clear association between time to recanalization, even in this selected cohort with groin puncture within 6 h of onset. Moreover, both advancing age and comorbidity were associated with poor outcomes. Shorter onset to treatment strategies, refinement of endovascular treatment, improved patient selection and neuroprotection remain important targets for improving outcomes. Recurrent stroke or other stroke-related complications may occur post-EVT and affect 90-day outcome negatively, emphasizing the importance of a comprehensive stroke follow-up in order to maintain the early favorable outcomes over time.^23^

We included patients with oral anticoagulants (19%), largely explaining the lower proportion receiving tPA in RSEVAS.

### Strengths

RSEVAS is a nationwide database reflecting current clinical practice and outcomes based on real-world data with excellent coverage. We consider the risk of selection bias to be low.

### Limitations

This study has several limitations: (1) With 18.7% loss to follow-up, there is a risk of attrition bias. sICH was more common in not followed up, occurring in 15/189 (8%) compared to 4.4% in followed up subjects, but otherwise no baseline, procedural, or early outcome data differed between groups, indicating that the effect of attrition on functional outcome should be limited. For that reason, we chose to show results both omitting these subjects from analysis and extrapolate functional outcome in followed up survivors to survivors lost to follow-up, and including loss to follow-up. (2) Comparisons to HERMES were made to aggregated data only and diffences in baseline data between RSEVAS and HERMES were unadjusted for. (3) Recanalization rates and other radiological outcome variables were evaluated by the treating interventionalist and not by core lab assessment which may affect results, presumably in a positive direction.^24^ (4) Functional outcome is self-reported and may be both over- and underestimated. However, self-reported data have shown good concordance with objectively assessed 90-day mRS.^25^

## Conclusion

In our large real-world national data set, patients treated with EVT differed to patients in the RCTs in several baseline factors including distribution of vascular occlusion site. However, despite the differences, the overall outcome of EVT in our Swedish cohort appeared to well match the pivotal trial findings.

## Supplemental Material

sj-pdf-1-ine-10.1177_15910199211073019 - Supplemental material for Survival and functional outcome following endovascular thrombectomy for anterior circulation acute ischemic stroke caused by large vessel occlusion in Sweden 2017–2019*–*a nationwide, prospective, observational study Click here for additional data file. Supplemental material, sj-pdf-1-ine-10.1177_15910199211073019 for Survival and functional outcome following endovascular thrombectomy for anterior circulation acute ischemic stroke caused by large vessel occlusion in Sweden 2017–2019*–*a nationwide, prospective, observational study by Teresa Ullberg, Mia von Euler, Johan Wassélius, Per Wester and Fabian Arnberg in Interventional Neuroradiology
